# Biodegradation of high concentrations of benzene vapors in a two phase partition stirred tank bioreactor

**DOI:** 10.1186/1735-2746-10-10

**Published:** 2013-01-15

**Authors:** Ali Karimi, Farideh Golbabaei, Masoud Neghab, Mohammad Reza Pourmand, Ahmad Nikpey, Kazem Mohammad, Momammad Reza Mehrnia

**Affiliations:** 1 Department of Occupational Health, School of Public Health and Nutrition, Shiraz University of Medical Sciences, Shiraz, Iran; 2 Department of Occupational Health, School of Public Health, Tehran University of Medical Sciences, Tehran, Iran; 3 Pathobiology Department, School of Public Health, Tehran University of Medical Sciences, Tehran, Iran; 4 Department of Occupational Health, School of Public Health, Qazvin University of Medical Sciences, Qazvin, Iran; 5 Department of Epidemiology and Biostatistics, School of Public Health, Tehran University of Medical Sciences, Tehran, Iran; 6 Biotechnology Group, School of Chemical Engineering, University College of Engineering, University of Tehran, Tehran, Iran

**Keywords:** Two phase partition bioreactors, (TPPBs), Benzene, Silicon oil, Biotreatment

## Abstract

The present study examined the biodegradation rate of benzene vapors in a two phase stirred tank bioreactor by a bacterial consortium obtained from wastewater of an oil industry refinery house. Initially, the ability of the microbial consortium for degrading benzene was evaluated before running the bioreactor. The gaseous samples from inlet and outlet of bioreactor were directly injected into a gas chromatograph to determine benzene concentrations. Carbone oxide concentration at the inlet and outlet of bioreactor were also measured with a CO2 meter to determine the mineralization rate of benzene. Influence of the second non-aqueous phase (silicon oil) has been emphasized, so at the first stage the removal efficiency (RE) and elimination capacity (EC) of benzene vapors were evaluated without any organic phase and in the second stage, 10% of silicon oil was added to bioreactor media as an organic phase. Addition of silicon oil increased the biodegradation performance up to an inlet loading of 5580 mg/m^3^, a condition at which, the elimination capacity and removal efficiency were 181 g/m^3^/h and 95% respectively. The elimination rate of benzene increased by 38% in the presence of 10% of silicone oil. The finding of this study demonstrated that two phase partition bioreactors (TPPBs) are potentially effective tools for the treatment of gas streams contaminated with high concentrations of poorly water soluble organic contaminant, such as benzene.

## Introduction

Benzene is a component of gasoline and aviation fuels and is extensively used in industrial syntheses. It is frequently found as a contaminant in soil, water, and air from many industrial processes and as a result of storage tank and pipeline leakage, accidental spills, and improper waste disposal practices [1].

Benzene is classified as one of the main pollutants by the US environmental protection agency (EPA) because it is a common organic contaminant in oxygen-limited soils and groundwater. There is considerable interest on adverse health effects and cancer-risk levels for exposures to benzene. Similarly, common components of gasoline such as benzene are well-known carcinogens in both animals and humans [2]. Due to the toxicity of benzene, elimination of this compound from waste gas streams is essential in order to meet regulatory emission standards [3].

To date, a number of highly efficient physical and chemical gas cleaning techniques have been developed for removal of various compounds from industrial waste gases. Chemical methods are capable of removing a broad spectrum of compounds. However they have the disadvantage of energy consumption and/or consumption of chemicals. Similarly, in physical methods the pollutants are not destroyed. Solid adsorbents or liquids have to be regenerated and often a new pollutant is created. As a cost-effective and environmentally safe alternative, biological techniques, using indigenous bacteria have been successfully applied for the treatment of waste gases contaminated with volatile organic compounds (VOCs) [4-6].

The efficacy of more traditional bioreactor configurations, such as biofilters and biotrickling filters, is often limited by inherent design limitations. This, in turn, confines their application to the treatment of only very dilute waste gases. Common problems encountered in these designs include airflow channeling, increased pressure drop, drying of the filter bed, low rates of substrate and oxygen mass transfer, and inhibition by toxic substrates [7-10]. The technology of two phase partitioning bireactors (TPPBs) was originally adapted from its bioreactor predecessors to address these limitations. They allow highly concentrated waste gases to be treated effectively comparable with physicochemical methods [6].

The present study examined benzene biodegradation rate by a mixed culture of suspended cells in a stirred tank bioreactor under normal conditions of pressure and temperature. Additionally, the effects of substrate concentration and cell growth as well as the effect of addition an immiscible organic phase on biodegradation rate of benzene were studied. Finally, the roles of this organic phase on the treatment of high concentration of benzene in industrial waste streams were evaluated.

## Materials and methods

### Bioreactor setup

**Figure 1:**
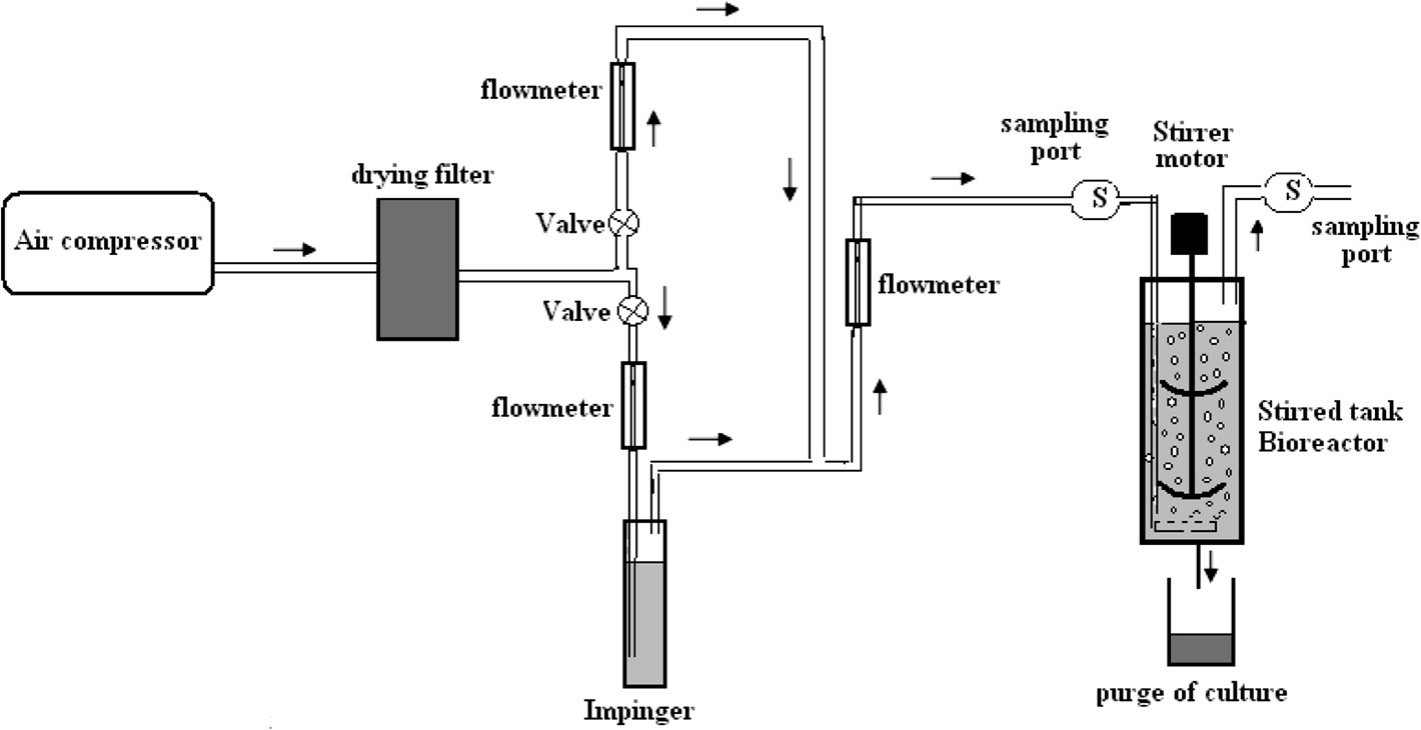
Schematic view of stirred tank bioreactor used in this study.

Figure 1 illustrates the setup of the bioreactor. As shown, the system consists of a 2.36 liter glass stirred tank, designed and used for all biodegradation activities. The bioreactor had an internal diameter of 10 cm and an operating volume of 1.77 liter (75%). The bioreactor was fitted with two dual Rushton turbine impellers, and lower impeller located 5 cm from the semi-circular base of the vessel. The bioreactor was also equipped with 4 equidistant baffles of 1 cm width to enhance liquid-liquid mixing and prevent vortex condition. Normal air introduced to dynamic generation system of benzene air stream. Introduced air was divided into two parts. A slow stream of air was bubbled through impinger (glass vial) containing pure benzene (Merck Co, Germany) and the second stream was bypassed. Both streams were mixed together again and metered with flow meter. By adjusting the flow rates of two streams, various concentrations of benzene could be produced. Known concentration of benzene in air stream was supplied to the system through an orifice sparger containing 9 orifices with total open area of 0.28 cm^2^, located below the lower impeller. The experiments were performed at room temperature and atmospheric pressure. An aeration rate of 1 l/min was tested in agitation speed of 400 rpm. The experimental conditions were selected in order to generate normal flow patterns inside the tank.

### Microorganism and mineral nutrients

The activated sludge of an oil industry refinery house was sampled based on the assumption that it contained benzene-degrading microorganisms and used as microbial consortium. Under sterile condition, the bioreactor wasrun with 75% working volume of mineral nutrient and microbial consortium with a proportion of 3:1 (vol). The nutrient medium consisted of: 1 g/L KH_2_PO_4_, 1 g/L K_2_HPO_4_, 0.2 g/L MgSO_4_, 1 g/L NaCl, 1 g/LKNO_3_ as well as trace elements that comprised of: 26 mg/L CaCl_2_.2H_2_O, 5.5 mg/L EDTA Na_4_(H_2_O)_2_, 1.3 mg/L FeCl_3_.4H_2_O, 0.12 mg/L CoCl_2_.6H_2_O, 0.1 g/L MnCl_2_.2H_2_O, 0.07 mg/L ZnCl_2_, 0.06 mg/L H_3_BO_3_, 0.025 mg/L NiCl_2_.6H_2_O, 0.025 mg/L NaMoO_4_.2H_2_O and 0.015 mg/L CuCl_2._2H_2_O [10].

The ability of the microbial consortium for degrading benzene was evaluated before running the bioreactor. For this purpose, 60 ml of the mineral solution was poured in 300 ml bottles as nutrient. The reminder 240 ml of headspace guaranteed sufficient air for aerobic degradation. Thereafter, benzene at a concentration of 100 mg/L was added to the test bottles as the sole carbon and energy source. All bottles were incubated at a temperature of 28°C on a rotary shaker at 90 rpm in a dark environment. The ability of samples for degrading benzene in head space and solution was evaluated by a gas chromatograph (Varian CP-3800) equipped with an FID detector. A similar flask, which contained 1% of KCN as a blank sample were prepared for control by benzene loss from volatilization and diffusion from septum. In each cycle, dissolved oxygen (DO) was measured with DO meter (model YK- 2001 DO).

### Organic phase selection

The organic solvent used in the two-phase partitioning bioreactor system should not be used as a substrate by the microorganism to ensure that the benzene acts as sole carbon source [11]. A non-aqueous phase suitable for use in TPPBs should be inexpensive, readily available, immiscible in water, nonbiodegradable, biocompatible (i.e. non-toxic to the microbial community) and should exhibit a high affinity to the limiting substrates in order to increase their mass transfer [12,13]. In recent studies most investigators have selected silicon oil rather than hexadecane, tetradecane, 1-decanol, diethyl sebacate, and 2-undecanone as the best non-aqueous phase for the treatment of VOCs in a stirred-tank bioreactor [13-15].

Silicon oil was the liquid partitioning phase, as it is non-bioavailable to microorganisms and can therefore, be used with the benzene degrading bacterial consortium. It has been reported to be non-toxic and non-biodegradable organic phase [16]. In addition, it is used as the sequestering phase in TPPBs by a number of other authors [3,13,16].

The volume proportion of Silicon oil as an organic phase was selected based on a primary examination in our previous study. Therefore the most optimal fraction of silicon oil in terms of oxygen transfer and total power consumption rate was found to be 10 percent [16].

### Analytical methods

To determine the removal efficiency and elimination capacity of the bioreactor, the gaseous samples were collected from the two sampling ports placed just before the inlet and after the outlet of bioreactor. Samples were directly injected into a gas chromatograph fitted with a flame ionizing detector and a fused silica capillary column (0.53 mm I.D., 50 m length, 0.25 μm film thickness, CP-Sil 8 CB). The carrier gas was helium at a flow rate of 30 ml/min. The injector, column, and detector temperatures were set at 200, 130, and 210°C, respectively. The concentration of CO_2_ at inlet and outlet of the bioreactor were measured with a CO2 meter (Hotek technologies Inc, USA). By determining the difference between inlet and outlet concentrations of CO_2_, the carbon content of CO_2_ was calculated and by dividing it by carbon content of biodegraded benzene(C-CO_2_/C-benzene biodegraded), the percentage of C-mineralized was determined.

For measuring benzene concentration in liquid phase, when the bioreactor turned off and the organic phase separated and raised up, 50 ml of silicon oil was sampled. Then, the sample was centrifuged at 3500 rpm for 10 min for complete separation of organic and aliquots phases. A sample of 20 μl of organic phase was immediately injected to GC/FID and concentration of benzene was calculated by comparing the produced peak area with standard curve.

Mixed liquid samples were periodically withdrawn and centrifuged at 2000 rpm for 10 min to separate the cells, aqueous phase and organic phase. The organic phase and aqueous phase removed from sediment wet biomass, then the wet biomass measured by gravimetric method.

### Operational parameters of bioreactor

The continuous operation of bioreactor was guaranteed by replacing 5-10% (vol) of culture medium described above. Meanwhile the proportion of silicon oil was kept constant (10%) by returning or adding the necessary amount to bioreactor medium. The RE and EC of bioreactor at various conditions were studied. The performance of waste gas treatment systems is usually represented by the following equations [3,17]:

Removal efficiency (RE):

(1) $$\mathrm{RE\%}=\frac{C_{\mathrm{in}}-C_{\mathrm{out}}}{C_{\mathrm{in}}}\times 100$$

Elimination capacity (EC):

(2) $$\mathrm{EC}\left[\mathrm{mg}/m^{3}/\mathrm{hr}\right]=\frac{Q\left(C_{\mathrm{in}}-C_{\mathrm{out}}\right)}{V}$$

Where Q is the gas flow rate (m^3^/h), V is the volume of the bioreactor medium (m^3^) C_in_ and C_out_ are the inlet and outlet pollutant concentrations (mg/m), respectively.

## Results

**Figure 2:**
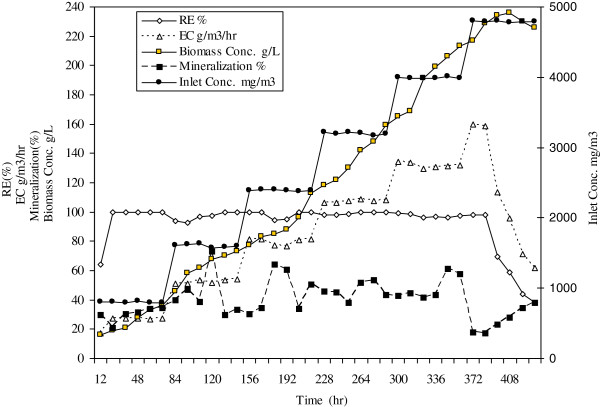
Continuous operation of bioreactor in the absence of silicon oil as organic phase.

**Figure 3:**
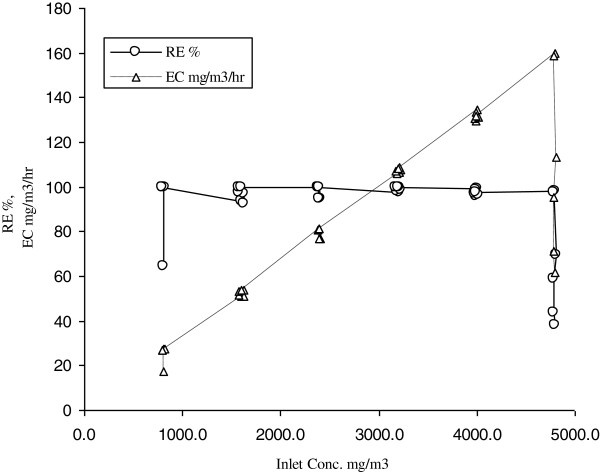
Removal Efficiency(RE) and elimination capacity(EC) of benzene as function of inlet concentration in the stirred tank bioreactor in the absence of silicon oil.

Benzene degradation rate by bacterial consortium were studied at different inlet concentration levels to reach optimized conditions. Figure 2 and Table 1 show operational parameters of biotreatment of benzene over the continuous operation without addition of silicon oil as organic phase. The removal efficiency and removal capacity of gaseous benzene were evaluated at inlet concentrations ranging from 794 mg/m^3^ to 4804 mg/m^3^ for 432 hours. The results revealed that an increasing trend in bioreactor performance exists up to a concentration of 3988 mg/m^3^ and at this concentration the elimination capacity and removal efficiency were 132 g/m^3^/h and 98% respectively. Under the above mentioned conditions the rate of cell growth was also remarkable (Figure 2) meaning that benzene has good bioavailability in stirred tank bioreactor. During this period no oxygen limitation was noted, however, when the inlet loading exceeded 4000 mg/m^3^, and approached to 4800 mg/m^3^ the outlet concentration of benzene increased rapidly and the biodegradation rate encountered a significant loss (Figures 2 and 3). This phenomenon is due to the growth inhibition at high substrate concentration which prevents the effective biodegradation of contaminants.

**Table 1:** Operational parameters of stirred tank bioreactor during biodegradation of benzene in the absence of silicon oil

	**Inlet conc. mg/m**^**3**^	**Outlet conc. mg/m**^**3**^	**RE (%)**	**EC g/m**^**3**^**/hr**	**Mineralization (%)**	**Biomass g/L**
Min	794	0	38	18	17	16
Max	4804	2970	100	160	73	236
Average	2797	303	92	85	40	120
SD	1363	726	16	40	13	70

**Figure 4:**
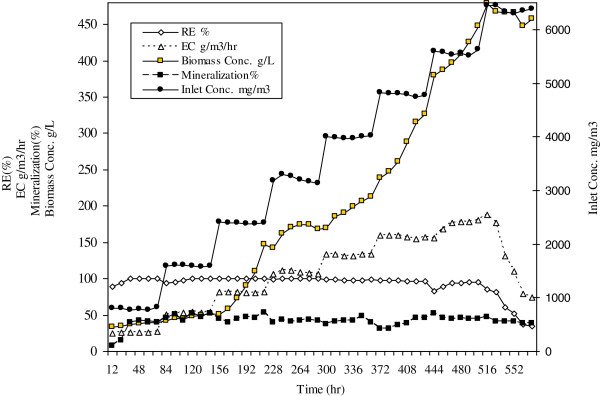
Continuous operation of bioreactor in the presence of 10% silicon oil as organic phase.

**Figure 5:**
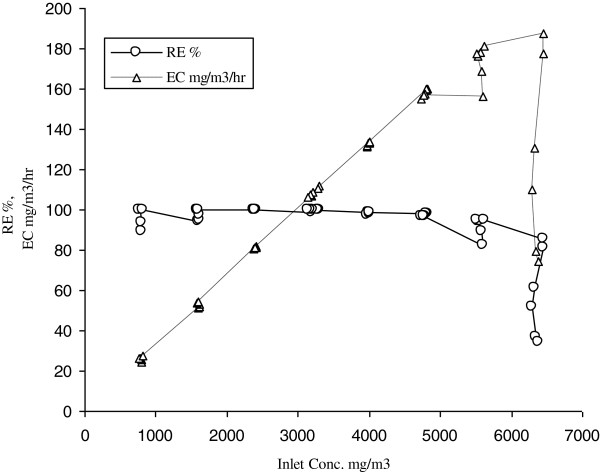
Removal Efficiency (RE) and elimination capacity(EC) of benzene as function of inlet concentration in the stirred tank bioreactor in the presence of 10% silicon oil.

Application of a second immiscible phase in the bioreactor can help to hinder the toxic effect of benzene on the microbial cells. Therefore in the next stage of this study, silicon oil was used to improve the bioreactor performance. The removal efficiency and removal capacity of gaseous benzene were also evaluated at inlet concentrations ranging from 798 mg/m^3^ to 6453 mg/m^3^ for 576 hours in the presence of 10% silicon oil as an organic phase. An increasing trend in bioreactor performance up to a concentration of 5580 mg/m^3^ was noted. At this concentration the elimination capacity and removal efficiency were 181 g/m^3^/h and 95% respectively. No oxygen limitation was expected because silicon oil has a high affinity to oxygen and is able to reserve the necessary amount of oxygen for biotreatment process. It was shown that presence of 10% silicon oil increased elimination capacity of benzene up to 38%. However when the inlet loading exceeded 5600 mg/m^3^, and approached 6400 mg/m^3^ the inhibition singswere evident. Consequently, the elimination capacity reduced significantly (Figures 4 and 5). It was found that the improvement of biotreatment process due to the presence of organic phase was limited itself and ultimately would be overcome by toxicity effect of substrate (Table 2).

**Table 2:** Operational parameters of stirred tank bioreactor during biodegradation of benzene in the presence of 10% silicon oil

	**Inlet conc. mg/m**^**3**^	**Outlet conc. mg/m**^**3**^	**RE (%)**	**EC g/m**^**3**^**/hr**	**Mineralization (%)**	**Biomass g/L**
Min	769	0	34	24	7	34
Max	6453	4185	100	188	53	479
Average	3589	420	93	107	42	210
SD	1825	964	15	50	8	152

## Discussion

Continuous stirred tank bioreactors and other suspended-growth bioreactors are appropriate for waste-gas treatment. In such systems, microorganisms constantly suspended a nutrient rich aqueous phase. The gas-phase pollutant, preferably often serves as the sole carbon and energy source for microbial growth [17].

The system initially was run with cell concentration of 16 g/l, and an initial loading of 794 mg/m^3^ benzene (Figure 2 and Table 1). As the cells were previously pre-adapted to benzene, no extra adaptation or lag period was observed in this experiment and the cell concentration began to increase almost immediately. However it was necessary to start with low inlet loading for the system to gradually acclimatize with higher concentrations.

Addition of silicon oil as a second liquid phase in the bioreactor media improved biotreatment of gaseous benzene from contaminated air stream. The maximum tolerable concentration of inlet loading in the presence of silicon oil was 5600 mg/m^3^. Perhaps the key element in the success of current system is the selection of an appropriate second phase. High performance of the system could be attributed to several factors. First, the solvent has a very high affinity for benzene (in fact benzene is infinitely soluble in silicon oil) providing a very effective means of VOC capture. This is in contrast to bio filters which often have difficulty absorbing hydrophobic VOCs by the small water layers associated with the packing and microbial film layers. Second, the solvent provides a sink for the VOC which can substantially act as a buffer (because of the high partition coefficient of benzene between silicon oil and water) to accommodate higher VOC loadings as they may occur, and also deliver VOCs already contained in the organic phase even during periods of low VOC application. Third, the system is completely mixed, allowing the total solvent volume to scrub the VOC, and the total reactor volume (i.e. all of the cells in the reactor) to be effective in degradation. Again, this is in contrast to biofilters that often do not equally and effectively use their entire reactor volume for VOC removal and degradation [18].

Despite the numerous advantages of TPPBs in environmental biotechnology, there is still a need for more knowledge on how to design and scale up these systems. In particular, the selection of the organic phase is a crucial step that must be carefully investigated as it determines both process costs and efficiency. Silicon oil was selected as the most suitable organic phase among the tested solvents due to its biocompatibility and resistance to microbial attack. Owing to these good characteristics of silicon oil, numerous authors have selected it as the organic phase for the degradation of toxic polycyclic aromatic hydrocarbons in TPPBs [15,19,20]. Additionally, microscopic observations showed that microbial cells were in direct contact with silicon oil. This suggests that benzene could have been taken-up by the microorganisms directly at the aqueous-organic inter-phase, which would further improve the efficiency of the bioreactor [19].

In our study, average pollutant mineralization also increased from 40 to 42% when silicon oil was present. This increase might be explained by the higher availability of O_2_ in the biphasic system (as the solubility of O_2_ is approximately 30 times higher in silicon oil than in water) [15].

The ECs obtained in the TPPB system (up to 181 g/m^3^/h) were higher than those of the best benzene removal rates yet reported in biofilters. For instance, Won HH et al. reported an EC of up to 100 g/m^3^/h in a biofilter packed with polyurethane foam [21]. Kim JO reported an average EC of 120 g/m^3^/h benzene in a biofilter [22]. Zhou Q recorded benzene EC of up to 11.5 g/m^3^/h with a biotrickling filter [23]. The high ECs reported in the TPPB were the consequence of the combined effect of the presence of silicon oil and the intense turbulence generated in the system, which both contributed to increase benzene mass transfer. Mechanical agitation, thus, greatly increased the gaseous/organic and organic/aqueous interfacial area for benzene transfer (by reducing the size of gas or organic bubbles) and provided homogeneous conditions in the system [19]. This represents a considerable advantage of stirred-tank bioreactors towards packed-bed systems, which are more sensitive to clogging and drying [24]. However, the potential of biphasic stirred tank reactors for large scale applications remains to be convincingly demonstrated [15].

Foaming in the TPPBs was unexpected due to the antifoaming properties of silicon oil. In other studies [20] authors have reported the appearance of foaming in silicon based TPPBs as a result of the high production of biosurfactants by Pseudomonas cultures under conditions of intense turbulence and incipient cell growth such as the conditions found in our bioreactor (400 rpm of stirring speed). Solvent emulsification as a result of the intense turbulence attained in the bioreactor could have reduced the antifoaming capacities of silicon oil as reported by Racz et al. [15]. In this study, the foaming that formed at the top of the organic phase was controlled by installing two foam breaking blades.

This study confirmed the potential of TPPBs for the treatment of gas streams contaminated with high concentrations of poorly soluble organic contaminant. However, besides being more efficient than conventional biofilters, TPPBs (commonly implemented in conventional fermentors) are more energy demanding due to the input required for stirring. Thus, more research on bioreactor design is needed to minimize energy consumption under conditions of intense agitation [15].

## Conclusion

Appreciable biodegradation rates of benzene from contaminated air streams using a well-mixed stirred tank bioreactor were achieved. By operating the bioreactor in a continuous mode, benzene removal efficiencies reached 95% with inlet loadings up to 181 g/m^-3^/h. The results showed that this reactor was flexible for high concentration of pollutant, especially by adding a second immiscible phase to the culture medium. Indeed, due to its high affinity for benzene, it seems that silicone oil increased the pollutant transfer rates from the gas phase. The pollutant was first trapped into the organic phase and then transferred to the aqueous phase driven by pollutant biodegradation in the aqueous phase.

## Competing interests

The authors declare that they have no competing interests.

## Authors’ contributions

AK and FG participated in the design of the study. AK, MRM and KM performed the statistical analysis. AK carried out the experimental studies. MN, AN and MRP helped to draft the manuscript. All authors read and approved the final manuscript.
